# Rehabilitation program for traumatic chronic cervical pain associated with unsteadiness: a single case study

**DOI:** 10.1186/1746-1340-16-15

**Published:** 2008-11-17

**Authors:** Danik Lafond, Annick Champagne, Rosalie Cadieux, Martin Descarreaux

**Affiliations:** 1 Département des sciences de l'activité physique, Université du Québec à Trois-Rivières, Trois-Rivières, Quebec, Canada; 2 Département de chiropratique, Université du Québec à Trois-Rivières, Trois-Rivières, Quebec, Canada

## Abstract

**Background:**

Neck problems are often recurring or chronic. After pain, unsteadiness and balance problems are among the most frequent symptoms reported by chronic neck pain (CNP) patients. Altered sensorimotor control of the cervical spine and sensorimotor integration problems affecting postural control have been observed in CNP patients. Very few data are available regarding the post-intervention effects of rehabilitation programs on postural control in CNP.

**Case presentation:**

This is a case study of a traumatic CNP patient (a 45-year old female) with postural unsteadiness who participated in an 8-week rehabilitation program combining therapeutic exercises with spinal manipulative therapy. Pre-intervention data revealed that the postural control system was challenged when postural control sensory inputs were altered, particularly during the head-extended-backward condition. Post-intervention centre of pressure measurements indicated a drastic reduction in postural sway during trials with changes in neck orientation.

**Conclusion:**

This case report indicates that an 8-week rehabilitation program combining therapeutic exercises with spinal manipulative therapy may have had an effect on improvement of postural control in a trauma CNP patient with unsteadiness. These results warrant further studies to investigate the relationships between pain amelioration, sensorimotor control of the cervical spine, muscle fitness and postural steadiness.

## Background

Neck disorders are among the most common and costly health complaints in industrial countries. Lifetime neck pain prevalence is 66% [1], and recurrent pain or episodes lasting more than 6 months have been reported in 14% of the adult population [2]. After pain, unsteadiness and balance problems are among the most frequent symptoms encountered by chronic neck pain (CNP) patients [3]. For instance, quantitative posturography studies have discerned increased postural sway in CNP compared to healthy subjects [4-6].

Postural steadiness and balance involve proprioceptive, vestibular and visual postural control subsystems. Cervical proprioceptive afferences play an important role in postural control by providing information regarding head position and displacements relative to the trunk [7]. Previous work has shown that modifying neck position challenges the postural control system, in both healthy and CNP subjects [8-10]. Kogler et al. [9] found that changes in neck position elicited more postural sway in neck pain subjects with vertigo compared to healthy controls. Neck muscle afferents enable the central nervous system to locate the head's orientation relative to the trunk and are linked to the vestibular system [11,12]. It is hypothesized that postural unsteadiness in CNP could result from a mismatch between modified neck proprioceptive afferences and normal vestibular afferences [4-6]. Altered sensorimotor control of the cervical spine has also been observed in CNP patients with increased neck joint repositioning errors [13-15]. In CNP cases, such disturbances are believed to be a consequence of aberrant cervical proprioceptive inputs or changes in sensorimotor integration. Modulated cervical sensorimotor control in neck pain is thought to occur via several mechanisms, including variations in fusimotor drive impacting muscle spindle sensitivity and modifying cortical representation of cervical afferent input [16-18] as a result of pain, muscle dysfunction and inflammation. Afferences from both labyrinth and neck muscle spindles converge to vestibular nuclei and evoke adaptive postural responses with head movement control strategies [11,12]. Gdowski and McCrea [19] have demonstrated that neck proprioceptive afferences contribute to the shaping of vestibular nucleus outputs, endowing postural steadiness. As a consequence of cervical muscle pain, impaired proprioceptive afferences could elicit mismatching between neck proprioceptive afferences and those from the normal vestibular system, resulting in sensorimotor integration disturbances affecting postural control, as observed in CNP patients. Armstrong et al. [20] pointed out that articular receptors of the cervical spine may complement muscle spindles in the position sense, and damage in mechanoreceptors of the cervical spine could contribute to the pathomechanism of neck pain. Muscle inhibition, muscle atrophy and increased muscle fatigability could also contribute to sensorimotor disturbances in CNP [21,22]. These factors seem to support the value of strengthening exercises such as therapeutic rehabilitation in neck pain patients.

The management of cervical sensorimotor control impairments associated with CNP may include strategies, such as exercises aimed at improving cervical proprioception and decreasing neck pain and disability. Therapy involving stretching or strengthening exercises could reduce pain and improve function in CNP, even though the evidence is still limited [23,24]. Recently, Jull et al. [25] found that proprioceptive exercises induced greater changes in the joint position sense than cranio-cervical flexion-based exercises. Treleaven [13,26] proposed a multimodal approach, including conventional physiotherapy as well as tailored oculomotor, proprioceptive and balance exercises to retrain sensorimotor control in CNP patients. On the other hand, manipulation when combined with exercises is more effective than manipulation alone in the treatment of neck pain [27-29].

To date, very few data are available regarding the post-intervention effects of rehabilitation programs on postural control in CNP patients with associated unsteadiness. The current paper represents a case study of traumatic CNP in a patient who participated in an 8-week exercise therapy program designed to retrain the neck/shoulder muscles and sensorimotor control of the neck. The rehabilitation program chosen combined exercise with spinal manipulative therapy. This study emphasizes the effect of intervention on postural steadiness.

## Case presentation

### History

Ms. X, a 45-year-old elementary school music teacher, reported that she had a traumatic neck and dorsal spine injury 2 years ago. It was diagnosed as cervicalgia and dorsalgia. She got up from a squatting position and hit her head under a steel box fixed on a wall 3 feet from the ground. She felt immediate bilateral neck, dorsal and lumbar pain and stiffness, and also reported blurred vision and nausea. The next day, she visited her physician where cervical, dorsal and lumbar X-rays were taken. No particular lesion could be identified by X-rays. Six months later, she was scheduled for CT and bone scans of the cervico-thoracic spine that once again did not lead to any specific diagnosis with the exception of moderate degenerative disc disease at T8 and T9. Her cervico-thoracic spine pain had persisted since then, accompanied by pain radiating to the right shoulder. The patient also reported moderate restriction of her cervical range of motion and intermittent occipital headache, particularly when neck pain was exacerbated. Her symptoms were increased by sustained neck positions, computer work for several minutes and sitting in a car for prolonged periods as driver or passenger. She also reported insomnia as a result of neck pain. She was off work for 15 months after the injury and returned to work progressively in the last 18 months, on a part-time basis. The patient received physical therapy during the first 18 months after her injury. Before consulting for exercise therapy, she received chiropractic treatments (mainly spinal manipulative therapy), twice a week for 3 months, to restore mobility of the cervical and dorsal spine. At that time, and based on the absence of any neurological signs, the patient was diagnosed as having "mechanical neck pain". Chiropractic treatments temporarily relieved her symptoms, but the pain and stiffness kept returning 48–72 h after spinal manipulative therapy. At the time of the first consultation in kinesiology (exercise therapy), moderate limitation in cervical range of motion was observed, with stiffness and tightness of the right upper trapezius muscle and bilateral trigger points in the medial scapular region. The patient reported baseline neck pain of 6/10 on the visual analogue scale (VAS) at the beginning of the intervention. She took non-steroidal anti-inflammatory drugs 2–3 times a week. She had no past history of neck pain and unsteadiness prior to the traumatic incident.

### Postural stability assessment

Sensorimotor control was assessed by posturography analysis a few days before and after the 8-week intervention program. Postural steadiness was measured on a force plate (OR6-2000, AMTI, Watertown, MA, USA). The patient was asked to stand barefoot on the force plate, with her feet in a narrow stance (feet side-by-side position), arms hanging at her sides and her head in a normal, forward-looking position. Outlines of her feet were traced to ensure that foot placement was constant across trials. Each trial lasted 30 s. A modified version of the Clinical Test of Sensory Interaction on Balance (mCTSIB) was used [30] to assess the relative contributions of 3 sensory inputs of the postural control system. In this case study, the mCTSIB involved 10 quiet standing trials (see Table 1), with a varying surface (firm and soft support) and visual input (eyes-open (EO) and eyes-closed (EC)). To reduce the contribution of the vestibular system or to exacerbate the mismatch between vestibular and neck proprioceptive inputs, 3 additional head positions were tested. The 3 neck positions were: maximum neck/head extension backward (EXT) and maximum lateral flexion of the neck to the right (RLF) and left sides (LLF). No trunk movement was allowed during the neck displacements. At the beginning of each trial, the patient was asked to perform neck movements within a comfortable limit and to maintain the position during the 30-s trial.

**Table 1:** Testing conditions during the modified Clinical Test of Sensory Interaction on Balance (mCTSIB)

Conditions	Vision	Surface*	Neck movements
1	Eyes open	Firm	Head neutral
2	Eyes open	Soft	Head neutral
3	Eyes open	Soft	Left lateral flexion
4	Eyes open	Soft	Right lateral flexion
5	Eyes open	Soft	Extension
6	Eyes closed	Firm	Head neutral
7	Eyes closed	Soft	Head neutral
8	Eyes closed	Soft	Left lateral flexion
9	Eyes closed	Soft	Right lateral flexion
10	Eyes closed	Soft	Extension

* Soft surface: a 10-cm thick layer of polyethylene foam placed on top of the platform.

Ground reaction forces and moments were recorded from the force platform. Analog signals were sampled at a frequency of 100 Hz and filtered with a zero-lag sixth-order Butterworth low-pass filter at 6 Hz of cut-off frequency. Details of data processing are reported elsewhere [31]. Mean centre of pressure (COP) speed (mm/s) and sway area (mm^2^) were calculated to characterize postural steadiness. COP speed was defined as total COP displacement divided by the total period. Minimal metrically-detectable changes (MMDC) for COP speed in both the antero-lateral (A/L) and medio-lateral (M/L) directions and COP sway area were calculated by the intra-class coefficient and standard deviation (SD) reported earlier [32]. For a 30-s trial in the EO and firm surface condition, the MMDC of COP speed were ± 1.73 mm/s and ± 0.71 mm/s in the A/L and M/L directions, respectively, and ± 80.1 mm^2 ^for COP sway area. These values served to detect clinically-significant changes in postural steadiness after the intervention. To the authors' knowledge, intra- and inter-session reliability and MMDC in COP measurements have never been tested in neck pain subjects.

### Exercise therapy

**Figure 1:**
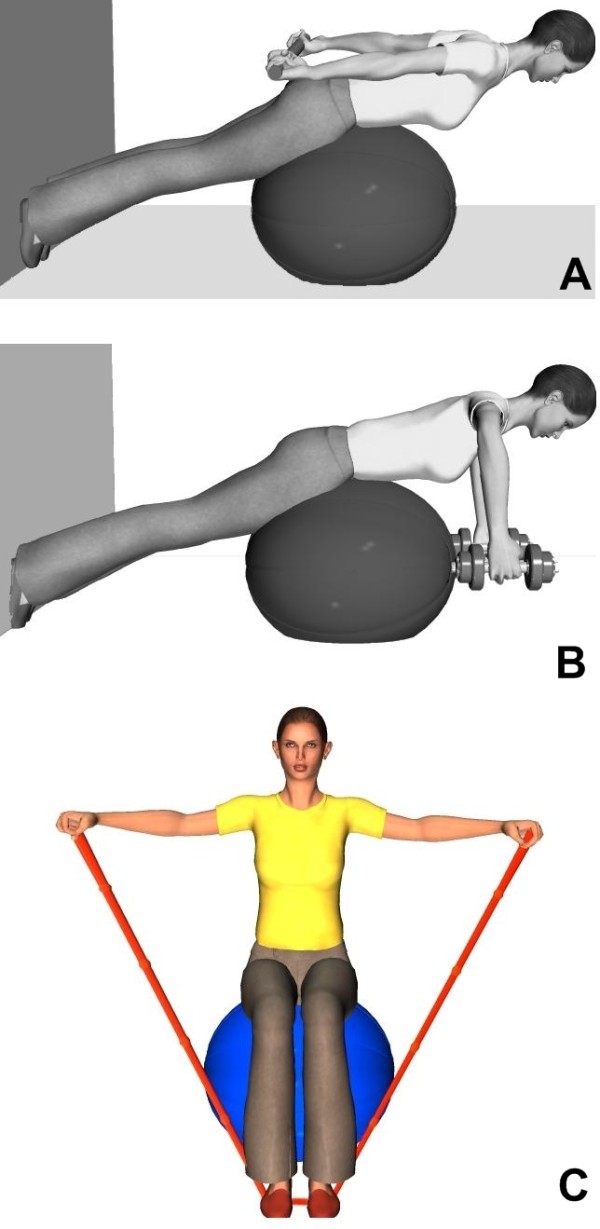
**Example of paraspinal, neck and shoulder girdle muscle-strengthening exercises.** A) Sorenson type exercise with isometric contraction to keep the shoulder in extension and the scapulas in adduction. B) Sorenson type exercise with thighs and hips supported on a Swiss ball. Isometric paraspinal contraction combining adduction/abduction of the scapulas. C) Isometric lateral shoulder raises with elastic resistance. The exercise could be performed sitting on a stable surface (e.g. a chair) or on a Swiss ball.

After the initial evaluation (18 months post-injury), the subject performed exercise training twice a week for 8 weeks. Each session, lasting 60 min, was supervised by an experienced kinesiologist. The exercise therapy program was aimed at improving neck muscle fitness and sensorimotor control of the cervical spine. It included:

▪ *Strengthening exercises*: with the head positioned against gravity to enhance isometric strength of the neck extensor muscles. Typical strengthening exercises for the paraspinal muscles and shoulder girdle muscles (upper and middle trapezius, rhomboids) are illustrated in Figure 1. These exercises were designed to increase sustained isometric effort tolerance of the neck muscles. Progression included unstable surface and escalating resistance.

**Figure 2:**
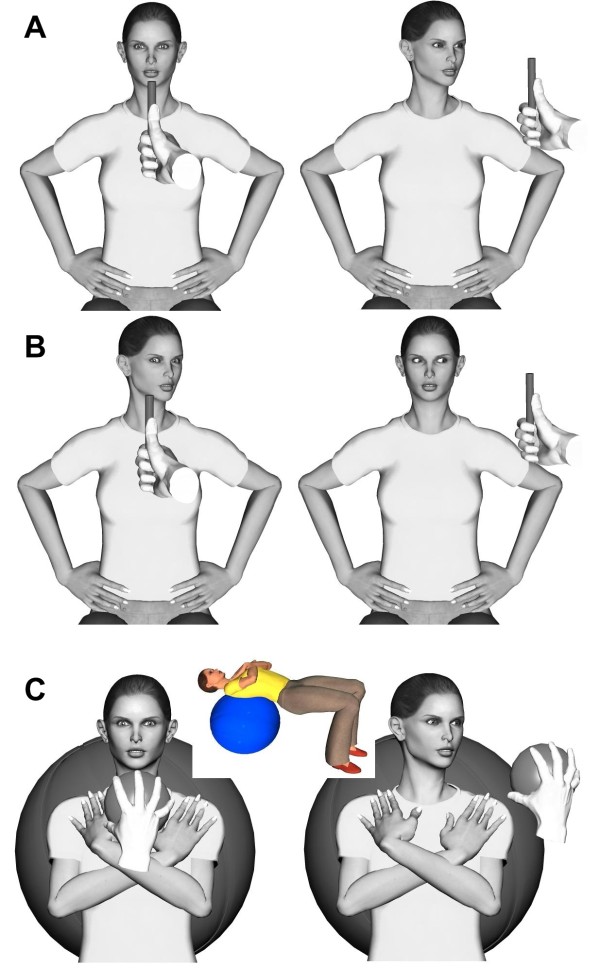
**Oculomotor and head/eye proprioceptive exercises.** A) Head-to-target or head movement following the target with the eyes in a neutral position. B) Eyes-to-target or eye movement following the target with different head positions. C) Head-to-target or head movement following the target with the eyes in a neutral position and the subject lying supine on a Swiss ball, with the head in a weight-dependent neutral position.

▪ *Oculomotor and head/eye exercises: *in the upright, sitting and supine positions. Eye tracking involved moving target exercises (Figure 2A) and eye/head coordination exercises (Figure 2B). Progression included increasing neck rotation amplitude, instability on a Swiss ball and augmenting neck muscle activity with the head in a weight-dependent position (Figure 2C).

**Figure 3:**
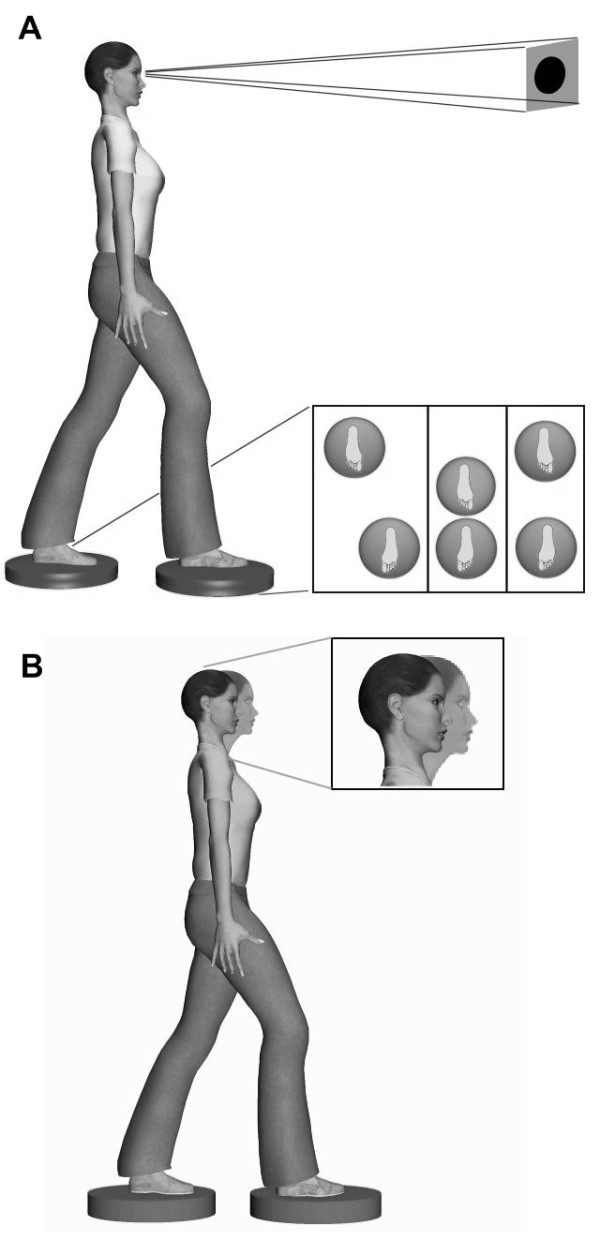
**Gaze stabilization and postural stability exercises.** A) Fixing a target during a challenging postural stability task. Feet in tandem positions increase postural constraints. B) Fixing a target during a challenging postural stability task combining movements of the neck/head.

▪ *Balancing exercises: *standing with *a *narrow stance, tandem stance and single leg stance. Progression included the use of foam under each foot to augment postural instability (Figure 3). Visual inputs were manipulated by focusing on a point 2 m away on the wall at eye level and under EO plus EC conditions. These exercises typically lasted 30 s.

▪ *Stretching exercises: *to sometimes reduce neck/shoulder stiffness and enhance neck range of motion.

### Spinal manipulative therapy

After ruling out all risk factors for major adverse events (vertebral artery dissection or the presence of major vertebral pathologies), the chiropractor initiated a series of treatments. Twice a week during the 8-week program, spinal manipulative therapy was applied to the patient's spine. The mobilization techniques and manipulated joints were chosen according to chiropractor clinical assessment that included the patient's history, physical examination as well as joint and muscle palpation. Treatment consisted of short-amplitude, high-velocity spinal manipulative thrust (chiropractic-diversified technique) on vertebral segments determined by manual palpation of joint restrictions and tenderness. Since pain on palpation was identified at the C2–C3 level on both sides, chiropractic adjustments were performed at this level either left or right, depending on the patient's pain tolerance.

### Effects of the rehabilitation program

Pain was the only clinical outcome formally monitored before and after the rehabilitation program. Prior to the program the patient reported significant pain and scored 6/10 on the VAS. Following the 8 week rehabilitation program, the patient scored 2/10 on the VAS. Associated neck disabilities were not assessed during the treatment period but the patient returned to work fulltime after a 24-month sick leave related to neck pain and disabilities. It was decided that the patient was able to return to usual working activities following what was described by the patient as a significant improvement in neck pain and related disabilities.

**Figure 4:**
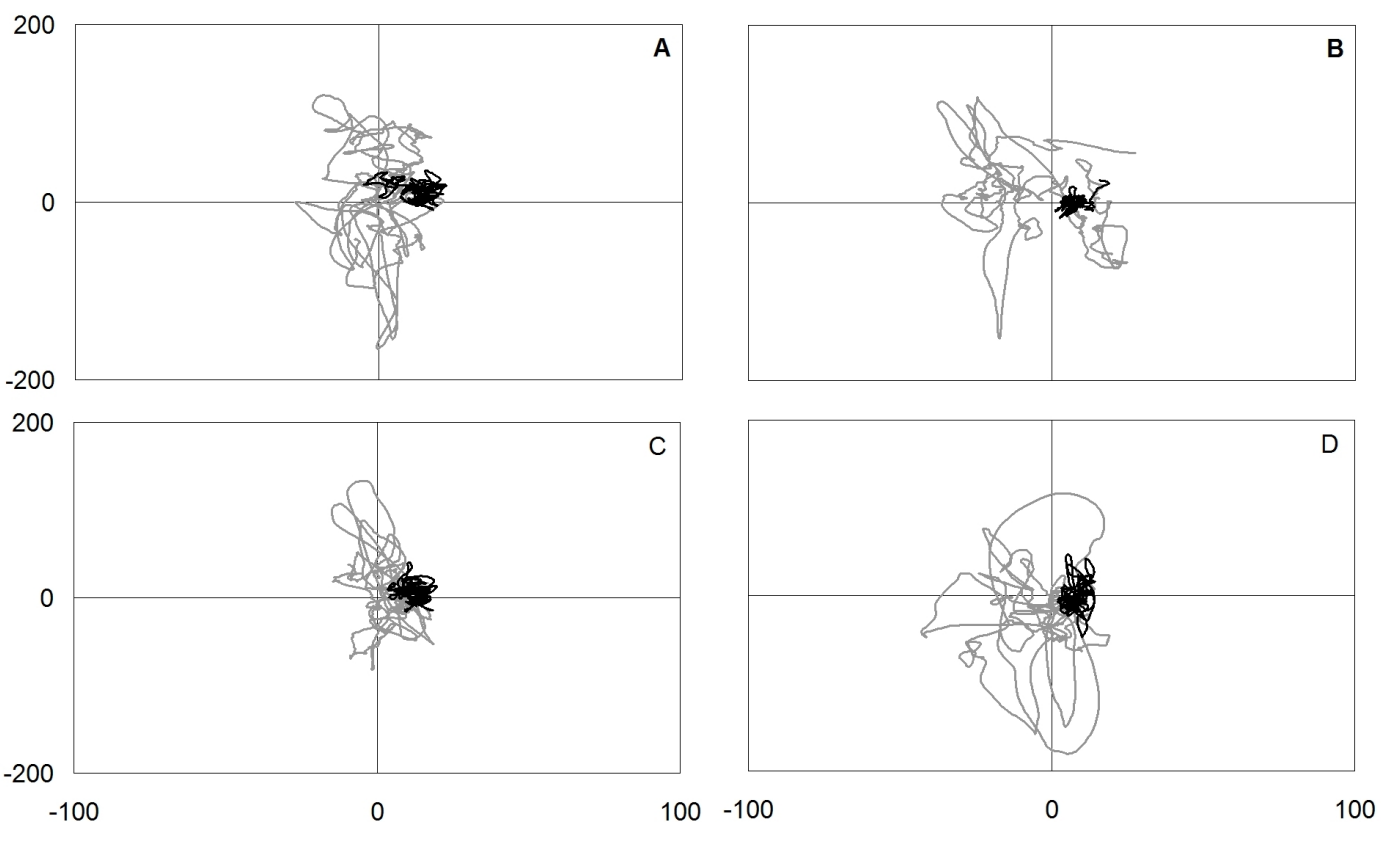
**Statokinesigrams (sway area) during eyes-closed conditions on a soft surface with different head positions: (A) head neutral; (B) left lateral flexion; (C) right lateral flexion; (D) extension.** (grey line): pre-intervention COP displacement; (black line): post-intervention COP displacement.

**Figure 5:**
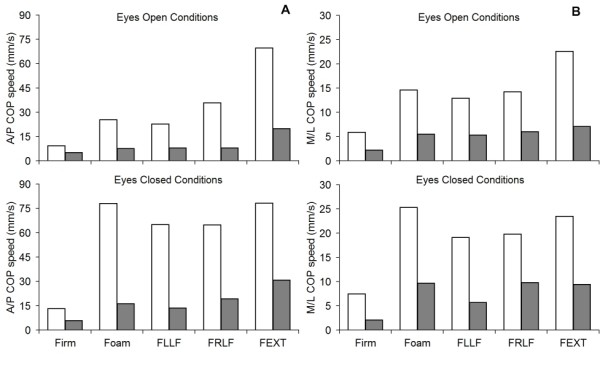
**Mean COP speed (mm/s) data during mCTSIB conditions before (blank) and after (black) the intervention in (A) the antero-posterior (A/P) and (B) medio-lateral (M/L) directions.** Firm = Firm support surface; Foam = Foam support surface; FLLF = Foam support surface with left lateral flexion; FRLF = Foam support surface with right lateral flexion; FEXT = Foam support surface with neck extension.

Pre- and post-intervention COP measures are shown in Figures 4 and 5. After 16 exercise sessions, the COP sway area decreased between 74.7% (EO, foam surface, LLF) and 95.4% (EC, foam surface, RLF). However, in condition 1 (EO, firm surface), the COP sway area increased from 86 mm^2 ^to 100.3 mm^2 ^(14.7%). This increment is well under the MMDC of ± 80.1 mm^2 ^and does not represent a clinically-significant change in postural steadiness. As depicted in Figure 5, COP speed values were reduced during all conditions after the exercise intervention in both the antero-posterior (A/P) and M/L directions. In the A/P direction, the decrease in COP speed ranged from 44.1% (EO, firm surface) to 79.1% (EO, foam surface, LLF). In the M/L direction, the diminution in COP speed ranged from 50.5% (EO, firm surface) to 72.0% (EO, foam surface, LLF). During condition 1 (EO, firm surface), the decline in COP speed represented a clinically-significant change in postural steadiness with -4.1 mm/s and -3.7 mm/s in the A/P and M/L directions, respectively.

## Discussion

The patient demonstrated postural unsteadiness hypothesized to be a consequence of traumatic CNP. Pre-intervention evaluation revealed that her postural control system was challenged when postural control sensory inputs were altered, particularly during the head-extended-backward condition. Compared to normative values, the COP data were well above those obtained in healthy adults.

To improve postural steadiness, we chose to use an intervention emphasizing strengthening and sensorimotor exercises combined with spinal manipulative therapy. Post-intervention COP measures indicated a drastic reduction in postural sway during trials with changes in neck orientation. Indeed, a greater decrease in postural sway was observed (in the range of 90–95% of the initial assessment) during balance conditions when sensory inputs were altered. For neutral head position conditions, post-intervention COP measures were close to the reference values obtained in young, healthy subjects in the same laboratory setting according to identical algorithm calculations (Table 2).

**Table 2:** Mean and standard deviation (mean ± SD) of centre of pressure (COP) variables calculated across 4 sensory conditions.

		mCTSIB			
		
COP variables	Direction	Condition 1	Condition 2	Condition 6	Condition 7
COP speed (mm/s)	A/P	7.6 (1.5)	10.8 (2.5)	9.9 (2.8)	10.6 (2.1)
	M/L	4.4 (1.2)	6.2 (2.2)	5.3 (1.7)	5.6 (1.7)
COP sway area (mm^2^)	--	181.4 (91.9)	261.1 (98.7)	187.2 (110.3)	220.2 (79.8)

Data were gathered from 30 subjects (age: 23.7 ± 3.8 years; weight: 66.3 ± 14.8 kg; height: 170.3 ± 11.8 cm)

Abbreviations: COP = centre of pressure; A/P = antero-posterior; M/L = medio-lateral.

Although the patient's postural steadiness improved, the information regarding clinical outcomes evolution is limited. One limitation of this case study was that neck pain was not systematically assessed during the intervention program. The patient reported a decrease in neck pain on the VAS from 6 to 2 post-intervention. She also disclosed a significant reduction in neck and upper trunk stiffness in the morning. Pain intensity is often considered as an outcome measure in therapeutic intervention studies. Nevertheless, the subjective rating of pain intensity in such investigations could be influenced by fluctuations in and the intermittent nature of neck pain. Several authors did not find a relationship between pain intensity and cervical kinesthetic sense [33-36]. However, Lee et al. [35] showed that pain frequency, not pain intensity, was associated with impairment of cervical kinesthetic sense. Further intervention and follow-up studies are needed to examine the relationship between the decline in pain intensity and frequency and the improvement in cervical kinesthetic sense, cervical function and postural steadiness.

Another limitation was that impairment of kinesthetic sense or sensorimotor control of the cervical spine (joint position error) was not assessed prior to and after the intervention program, and neither was oculomotor control. It is thus impossible to link the improvement of postural control to increased sensorimotor control of the cervical spine and oculomotor control. Previous work showed that proprioceptive exercises, similar to those prescribed in this study, enhance kinesthesia and position sense of the cervical spine in CNP subjects [25,37]. On the other hand, improvement in muscle force/endurance may have been responsible for the changes observed in postural stability [22].

Disability and quality of life questionnaires [38] are recommended in the assessment of CNP patients and could have been used in this particular case. Finally, the lack of follow-up assessments, owing to the fact that the patient was returned to work by her physician, should also be considered as a limitation of the present study.

## Conclusion

This case report indicates that an 8-week rehabilitation program combining therapeutic exercises with spinal manipulative therapy may have had an effect on improvement of postural control in a trauma CNP patient with unsteadiness. However, the amelioration of postural steadiness after an intervention program emphasizing strengthening and sensorimotor exercises deserves further investigation. Possible relationships between pain improvement, sensorimotor control of the cervical spine, muscle fitness and postural steadiness need to be explored.

## Consent

Written informed consent was obtained from the patient for publication of this case report and any accompanying images. A copy of the written consent is available for review by the Editor-in-Chief of this journal.

## Competing interests

The authors declare that they have no competing interests.

## Authors' contributions

DL and MD participated in the intervention and writing of the manuscript. DL, AC and RC undertook sensorimotor assessment and data analysis. MD performed all clinical evaluations. All authors have read and concur with the final manuscript. They also accept responsibility for its contents. The article has not been submitted or published elsewhere.
